# P-1285. Investigating Carbapenemase Genes Among Clinical Isolates of Gram-negative Organisms from the Philippines Using Whole Genome Sequencing

**DOI:** 10.1093/ofid/ofaf695.1473

**Published:** 2026-01-11

**Authors:** Twisha S Patel, Ze Qin Lim, Natascha May Thevasagayam, Song Qi Dennis Loy, Ma Tarcela S Gler, Gina De Guzman Betito, Jay Christian D Muere, Charlene R Siza, Olivia L McGovern, Fernanda C Lessa, Kalisvar Marimuthu, Oon Tek Ng

**Affiliations:** Centers for Disease Control and Prevention, Atlanta, GA; NCID, Singapore, Not Applicable, Singapore; NCID, Singapore, Not Applicable, Singapore; NCID, Singapore, Not Applicable, Singapore; Makati Medical Center, Manila, National Capital Region, Philippines; Makati Medical Center, Manila, National Capital Region, Philippines; Makati Medical Center, Manila, National Capital Region, Philippines; Centers for Disease Control and Prevention, Atlanta, GA; U.S. CDC, Atlanta, Georgia; CDC, Atlanta, Georgia; NCID, Singapore, Not Applicable, Singapore; National Centre for Infectious Diseases, Singapore, Singapore

## Abstract

**Background:**

Multidrug resistance among Gram-negative bacteria (GNB) is a serious global health threat, particularly in the Asia-Pacific region. A national surveillance program reported increasing rates of carbapenem resistance among GNB in the Philippines, ranging from 8%-18.9% in 2023, with metallo-β-lactamases being the most common mechanism of resistance. We used whole genome sequencing (WGS) to screen for carbapenemase genes and identify genetic lineages of carbapenem-non-susceptible (NS) clinical isolates collected from a private teaching hospital in Manila, Philippines.

**Figure 1.**
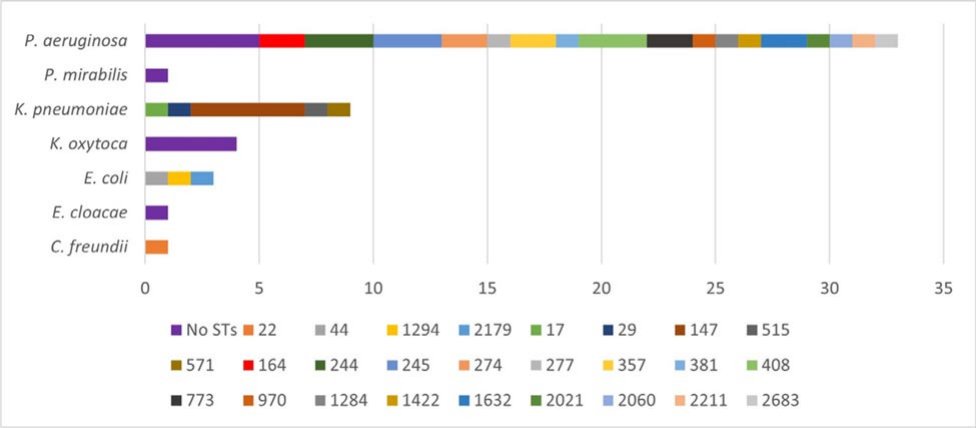
Distribution of ST by Organism Species

**Figure 2.**
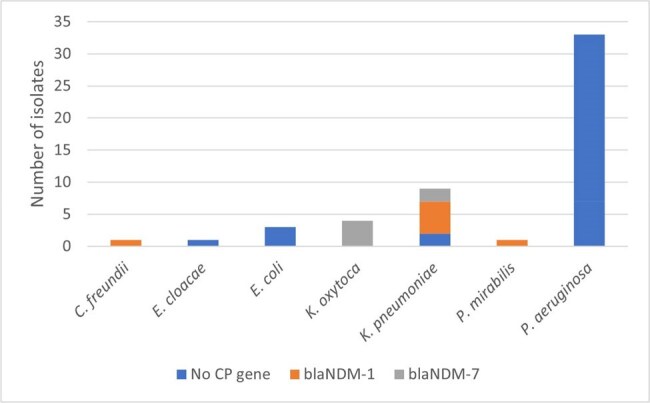
Distribution of Carbapenemase Genes Amongst Carbapenem-NS GNB

**Methods:**

All clinical isolates of carbapenem-NS GNB from hospitalized adults ( >18 years of age) in acute care wards were collected from March-November 2023. Following WGS and assembly, genomic species and sequence type (ST) were determined by MLST and Kraken, and AMRfinder was used to detect carbapenemase genes. Additionally, isolates were assessed for clonal and plasmid-mediated transmission of these genes.

**Results:**

Eighty-four carbapenem-NS isolates were identified during the study period, and 59 (70%) were sent for WGS. Of those, 52 isolates from 33 unique patients were found to have concordant species between clinical laboratory (MALDI-TOF) and genomic methods, with the majority being *Pseudomonas aeruginosa* (n=33), *Klebsiella pneumoniae* (n=9), and *Klebsiella oxytoca* (n=4). ST distribution is shown in Figure 1. Of the 19 carbapenem-NS Enterobacterales isolated from 16 patients, 13 (68.4%) had a carbapenemase gene detected with bla_NDM-7_ found in 6 (46.2%) and bla_NDM-1_ found in 7 (53.8%) isolates (Figure 2). No carbapenemase genes were identified in *P. aeruginosa*. Nine clonal clusters were determined, involving 22 isolates from 10 patients. Of the 13 carbapenemase-positive isolates found in 11 patients, four met criteria for potential plasmid-mediated transmission of a carbapenemase gene and were not part of a clonal cluster.

**Conclusion:**

Our investigation detected instances of both clonal and plasmid-mediated transmission of carbapenem resistance, with most overall occurrence caused by neither. The prevalence of carbapenemase genes among the tested carbapenem-NS Enterobacterales was high, with bla_NDM-7_ and bla_NDM-1_ being exclusively identified.

**Disclosures:**

All Authors: No reported disclosures

